# An Internet- and Kinect-Based Multiple Sclerosis Fitness Intervention Training With Pilates Exercises: Development and Usability Study

**DOI:** 10.2196/41371

**Published:** 2023-11-08

**Authors:** Andrea Tacchino, Michela Ponzio, Paolo Confalonieri, Letizia Leocani, Matilde Inglese, Diego Centonze, Eleonora Cocco, Paolo Gallo, Damiano Paolicelli, Marco Rovaris, Loredana Sabattini, Gioacchino Tedeschi, Luca Prosperini, Francesco Patti, Placido Bramanti, Elisabetta Pedrazzoli, Mario Alberto Battaglia, Giampaolo Brichetto

**Affiliations:** 1 Scientific Research Area Italian Multiple Sclerosis Foundation Genoa Italy; 2 Multiple Sclerosis Center IRCCS Foundation “Carlo Besta” Neurological Institute Milan Italy; 3 Vita-Salute University & Hospital San Raffaele Milan Italy; 4 Department of Neuroscience, Rehabilitation, Ophthalmology, Genetics, Maternal and Child Health University of Genoa Genoa Italy; 5 IRCCS San Martino Hospital Genoa Italy; 6 Neurology Unit IRCCS Neuromed Pozzilli Italy; 7 Department of Medical Science and Public health University of Cagliari Cagliari Italy; 8 Department of Neuroscience University of Padua Padua Italy; 9 Department of Basic Medical Sciences, Neuroscience and Sense Organs University of Bari Bari Italy; 10 Multiple Sclerosis Center IRCCS Don Carlo Gnocchi Foundation Milan Italy; 11 Uosi Multiple Sclerosis Rehabilitation IRCCS Istituto delle Scienze Neurologiche of Bologna Bologna Italy; 12 Department of Advanced Medical and Surgical Sciences University of Campania Luigi Vanvitelli Naples Italy; 13 Department of Neurosciences S. Camillo-Forlanini Hospital Rome Italy; 14 Department of Medical and Surgical Sciences and Advanced Technologies University of Catania Catania Italy; 15 IRCCS Centro Neurolesi “Bonino-Pulejo” Messina Italy; 16 Rehabilitation Service of Padua Italian Multiple Sclerosis Society Padua Italy; 17 Department of Life Science University of Siena Siena Italy; 18 Rehabilitation Service of Genoa Italian Multiple Sclerosis Society Genoa Italy

**Keywords:** exergame, Multiple Sclerosis Fitness Intervention Training, MS-FIT, Pilates, Kinect, multiple sclerosis, exercise, serious games, balance, mobile phone

## Abstract

**Background:**

Balance impairments are common in people with multiple sclerosis (MS), with reduced ability to maintain position and delayed responses to postural adjustments. Pilates is a popular alternative method for balance training that may reduce the rapid worsening of symptoms and the increased risk of secondary conditions (eg, depression) that are frequently associated with physical inactivity.

**Objective:**

In this paper, we aimed to describe the design, development, and usability testing of MS Fitness Intervention Training (MS-FIT), a Kinect-based tool implementing Pilates exercises customized for MS.

**Methods:**

MS-FIT has been developed using a user-centered design approach (design, prototype, user feedback, and analysis) to gain the target user’s perspective. A team composed of 1 physical therapist, 2 game programmers, and 1 game designer developed the first version of MS-FIT that integrated the knowledge and experience of the team with MS literature findings related to Pilates exercises and balance interventions based on exergames. MS-FIT, developed by using the Unity 3D (Unity Technologies) game engine software with Kinect Sensor V2 for Windows, implements exercises for breathing, posture, and balance. Feedback from an Italian panel of experts in MS rehabilitation (neurologists, physiatrists, physical therapists, 1 statistician, and 1 bioengineer) and people with MS was collected to customize the tool for use in MS. The context of MS-FIT is traveling around the world to visit some of the most important cities to learn the aspects of their culture through pictures and stories. At each stay of the travel, the avatar of a Pilates teacher shows the user the exercises to be performed. Overall, 9 people with MS (n=4, 44% women; mean age 42.89, SD 11.97 years; mean disease duration 10.19, SD 9.18 years; Expanded Disability Status Scale score 3.17, SD 0.75) were involved in 3 outpatient user test sessions of 30 minutes; MS-FIT’s usability was assessed through an ad hoc questionnaire (maximum value=5; higher the score, higher the usability) evaluating easiness to use, playability, enjoyment, satisfaction, and acceptance.

**Results:**

A user-centered design approach was used to develop an accessible and challenging tool for balance training. All people with MS (9/9, 100%) completed the user test sessions and answered the ad hoc questionnaire. The average score on each item ranged from 3.78 (SD 0.67) to 4.33 (SD 1.00), which indicated a high usability level. The feedback and suggestions provided by 64% (9/14) of people with MS and 36% (5/14) of therapists involved in the user test were implemented to refine the first prototype to release MS-FIT 2.0.

**Conclusions:**

The participants reported that MS-FIT was a usable tool. It is a promising system for enhancing the motivation and engagement of people with MS in performing exercise with the aim of improving their physical status.

## Introduction

### Background

Balance control impairments are common in people with multiple sclerosis (MS) [1] and may affect approximately 75% of people with MS during the course of the disease [1,2]. Weakness, spasticity, and fatigue are just some of the factors that cause balance disorders; moreover, central sensorimotor integration of visual, proprioceptive, and vestibular inputs is a critical aspect of balance control that may underlie dysfunction in static and dynamic balance [3,4]. Imbalance is usually characterized by the reduced ability to maintain position and to move toward the limits of stability and delayed responses to postural adjustments [1,4]. These abnormalities, together with other impairments and disabilities, often prevent people with MS from correctly and easily performing daily living activities and are considered as a primary fall risk in MS [5] with many adverse physical and psychological consequences (eg, injuries, fear of falling, and loss of autonomy) [6,7].

Mainly for these reasons, MS is associated with physical inactivity [8,9] that may lead to rapid symptom worsening and increased risk of secondary conditions (eg, fractures, high rates of depression, and fatigue). In turn, these may seriously affect participation in societal interactions and ultimately translate into reduced quality of life (QoL) [10]. Thus, balance exercises have increasingly been recommended in MS to control symptoms, enhance participation in daily activities, and improve QoL [11]. Balance exercises can be considered as the cornerstone of MS interventions [4].

Pilates is a popular alternative method for the maintenance and improvement of balance [12]. It is a precisely controlled form of exercise that uses stabilizing muscles of the body. Specifically, Pilates uses a holistic approach: concentration, control, centering, flowing movement, precision, and breathing are 6 fundamental principles; if correctly followed, they can improve flexibility, strength, core stability, muscle control, breathing, and posture and increase body awareness with less ground impact and joint stress [12,13]. These aspects and the possibility to perform exercises at the most convenient intensity levels (ie, difficulty adjusted to own level of conditioning) make Pilates an ideal approach to treat physical symptoms of MS [14]. More specifically, although high-quality studies are needed owing to the methodological flaw of most of the current literature, a recent meta-analysis showed that Pilates exercises might be an optional effective method to treat balance in people with MS [15].

In this context, the advancement of technology makes new alternatives available to deliver rehabilitative interventions in the form of internet- and game-based exercises (exergames) [16]. Playing exergames is a form of whole-body physical exercise delivered via commercial nonimmersive virtual reality [17] that requires users to complete the assigned tasks aimed at improving fitness and promoting an active lifestyle [18]. Exergames stimulate users to be more active, allowing repetitive practice and real-time interaction with feedback and tracking of progress, which enhance motivation, treatment adherence, and training outcomes [19-21]. Games that are specifically designed to address a problem or teach a certain ability have had a lot of success [22]. Researchers have demonstrated that exergames are comparable with traditional therapies and can be more fun and acceptable [23].

In the field of neurological diseases, a recent meta-analysis [24] explored the extent to which exergaming was superior to physical therapy or other forms of rehabilitation in improving balance dysfunction. Results were consistent with those of previously published meta-analyses and systematic reviews supporting the efficacy of exergames in the neurological setting [25-28]. A beneficial effect of exergames was found in stroke; Parkinson disease; and to a lesser extent, in MS, whereas conclusions on other conditions, such as mild cognitive impairment or Alzheimer disease, traumatic brain injuries, and myelopathies, were not possible. Furthermore, the effect size of exergames on balance significantly depended on the weekly frequency of sessions, single session duration, and overall intervention duration, whereas it was independent of the setting (supervised or home-based) and strategy (exclusive or add-on) of the intervention. Finally, exergames were found to be safe, with only mild to moderate musculoskeletal adverse events found in a minority of patients, with no significantly increased risk of falls [29].

Among the internet- and game-based technologies used for exercise training, the Kinect motion sensor (Microsoft Corp) constitutes a high-potential approach; it provides a precise markerless, full-body 3D motion tracker with real-time feedback about user performance and enables users to virtually interact hands-free with a computer system without needing a remote controller [30-35]. Lozano-Quilis et al [36] demonstrated the effectiveness of RemoviEM, a Kinect-based system offering people with MS an intuitive and motivating way to perform weight transfer and upper limb coordination exercises. Results showed great improvement in balance outcomes in the group using RemoviEM compared with the control group receiving the same types of exercises in a standard rehabilitative intervention. More recently, Molhemi et al [37] assessed the efficacy of a rehabilitative protocol including standing, walking, and weight shifting exercises delivered through a Kinect-based versus conventional training on the improvement of balance and mobility in people with MS. Both interventions improved balance and mobility; however, training with exergames was more efficacious in enhancing cognitive-motor function and reducing falls, whereas conventional exercises led to better directional control.

In the next years, new exergames will become available to the therapists, and it is hoped that these will allow for the integration in MS outpatient and at-home settings to optimize patient clinical outcomes and to ensure the continuity of care [38]. On the basis of the usability challenges faced by people with MS, programmers should develop user-friendly interfaces customized for people with MS, which is the main assumption for high acceptance, adherence, and effectiveness.

### Objective

The aim of this study was 2-fold: first, to design and develop a usable Kinect-based exergame implementing Pilates exercises appropriately adapted to MS requirements; second, to evaluate the usability in people with MS for future research and clinical use. An Italian network of experts in the field of MS rehabilitation was involved in the different phases of the study. The first steps were dedicated to literature revision and exercise identification, followed by prototyping based on co-design with MS health care professionals and people with MS. Usability testing was conducted on the final version of the tool.

## Methods

### Overview

This study consists of 2 parts: the design and development of an exergame implementing Pilates exercises and its customization for the needs of people with MS (part 1) and evaluation of its usability in terms of ease of use, playability, enjoyment, satisfaction, and acceptance (part 2).

The exergame was developed for asynchronous telerehabilitation purposes. The patient should be clinically supervised by a physical therapist for the evaluation of the progress of training and performances (Table 1). Moreover, for patients with neurological problems, no limitations in terms of session frequency and duration are considered; the more use, the higher the effect size of the exergame [24].

**Table 1:** Summary of the characteristics of Multiple Sclerosis Fitness Intervention Training.

Characteristic				Description
**Basic characteristics**				
	Targeted population		People with MS^a^ (adaptation needed for other clinical populations)	
	Exergame idea		The exergame is an internet game-based training system using Microsoft Kinect motion sensor technology It comprises games implementing Pilates exercises for breathing, posture, and balance	
	Target player		Individual	
	Behavior change		An exergame to enhance the motivation and engagement of people with MS in performing regular exercise	
	Clinical supervision		Physical therapist	
	Data shared with the user and clinician		Data are saved and stored in the hard disk and, if the tool is connected to the internet, uploaded to cloud Performances in terms of the level of correctness, gained internet-based coins, frequency and duration of exercise sessions, exercises performed or skipped, unlocked cities, and completed missions are given as feedback on the display screen during the execution of the game or through dedicated menus	
	Type of game		Pilates exercises for breathing, posture, and balance	
	Software version		Release 2.0	
**Game components**				
	**Exergame goal**			
		Physical components	Balance improvement Improve flexibility, strength, core stability, muscle control, and breathing Improve posture Increase body awareness Reduce ground impact and joint stress	
		Cognitive components	Cognitive improvement (however, no specific exercises stimulated cognition) Improve verbal and visuospatial memory, verbal fluency, and information processing speed Decrease cognitive fatigue Improve cognitive-motor, dual-task performances	
	Rules		Breathing: breathe in and breathe out in correspondence of the teacher’s instruction Posture: sitting or standing depending on the exercise requirements (a chair should be ready to use for the sitting exercises) Prompts: follow trajectories or arrows displayed in correspondence of the user’s avatar during the exercise execution	
	Game mechanics		The user interacts with the exergame by repeating the gesture of the Pilates trainer’s avatar The exergame also provides audio and visual feedback to the users while they are playing the games	
	Web environment		Travel to visit some of the most important cities around the world	
	Setting		Outpatient and at-home room environment with at least 3-m distance between the sensor and user	
	Hardware configuration		Minicomputer, monitor with a HDMI^b^ connector (minimum suggested size: 27 tTFT^c^), Microsoft Kinect Sensor version 2, and power lead controller	
	Sensors used		Microsoft Kinect Sensor version 2 (or new compatible commercial sensors such as Microsoft AzureKinect or MentorAge)	
	Estimated play time		30 minutes per day	

^a^MS: multiple sclerosis.

^b^HDMI: high-definition multimedia interface.

^c^TFT: thin film transistor.

### Ethical Considerations

The study was approved by the regional ethics committee of the principal investigator (Azienda Ospedaliera San Martino Genoa; n° 134/2018). All participants gave written informed consent before entry into the study in accordance with the revised Declaration of Helsinki [39].

### Part 1: Design and Development of a Pilates Exergame

#### Overview

**Figure 1:**
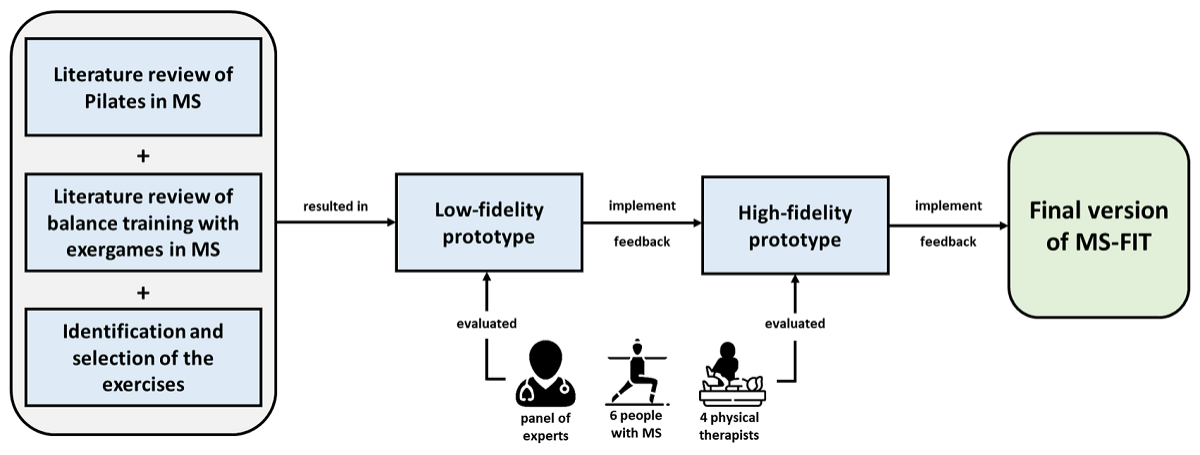
The app development process involving a literature revision on Pilates and balance training with exergames in multiple sclerosis (MS) and the identification and selection of the exercises to be implemented in MS Fitness Intervention Training (MS-FIT). A low-fidelity prototype and a high-fidelity prototype have been developed after evaluation by people with MS, physical therapists, and a panel of experts. Feedback was implemented to obtain the final version of MS-FIT. The icons in the figure have been modified from free licensed images from Flaticon [44].

Part 1 implements the user-centered design (UCD) approach [40-43], a user interface iterative design process that allows designers to gain the target user’s perspective. Outcomes of the UCD process are products with a high degree of usability. The UCD approach is composed of 4 phases: design, prototype, users’ feedback, and analysis [34]. All the study phases were performed between November 2018 and November 2019. The tool, customized for people with MS, was named MS Fitness Intervention Training (MS-FIT). Figure 1 presents the development process of MS-FIT.

#### Phase 1: Analysis of Existing Findings and Tool Design

During the first phase, a development team of 4 members, composed of 1 physical therapist (25 years of experience in neurological disease, including MS, and certified Pilates instructor), 2 game programmers (5 years of experience in the Unity 3D game engine), and 1 game designer (10 years of experience in game design, game theory, innovative virtual reality, and computer vision techniques), provided the design of MS-FIT. It implements some decisive elements that could enhance usability and user experience such as accessibility, challenge, reward and feedback, skill development, and difficulty adjustment [45,46].

Potential core game ideas were generated by integrating the team knowledge and results from a literature review about Pilates in MS and balance interventions in MS with exergames [29,30,35-38,40-43,45-56]. The Pilates exercises were identified for feasible implementation and safe execution in a digital tool [46]. The exercises aimed to train *breathing* (exercises for abdominal, costal, and sternal breathing perception; sitting and kneeling Mermaid exercises; and sitting and standing arm circles exercises able to increase the respiratory capacity; improve mobilization of the rib cage; and align and stabilize the trunk, backbone, head, pelvis, and shoulder), *posture* (eg, arch-and-curl and spine stretch exercises for the correct alignment of the trunk, backbone, head, pelvis, and shoulder in neutral position and during flexion extension; spine twist exercises to correctly prepare the trunk and upper limbs to gait scheme; exercises for self-correction of posture; and elephant exercise to stretch the backbone and hamstring muscles), and *balance* (eg, side-to-side, hip circle, side leg kick, 1-leg circle, and single leg extension exercises for correct weight transfer and monopodalic support). To ensure that the exergame was appropriate for each potential user, exercises at different levels of accessibility were considered (eg, some exercises are executed while seated). The game-based training program underwent critical appraisal by the physical therapist.

Multimedia Appendix 1 includes the description of Pilates exercises considered to be clinically beneficial to improve physical (ie, balance, mobility, and fatigue) and cognitive performances and QoL and easily implementable in MS-FIT [49].

Besides the identification and selection of the exercises, the design model phase took into account other essential factors such as rules and gameplay, options, levels of challenge, game plot and story, and transfer into real life [46,57].

#### Phase 2: Prototyping

During the second phase, the Pilates exercises were implemented in a low-fidelity prototype. The characteristics of MS-FIT are presented in Table 1.

The developers used the Unity 3D game engine software with Kinect Sensor V2 for Windows, a markerless tool usually adopted in different research scenarios as a low-cost device alternative to time-of-flight sensors (eg, SR-4000 CW10 by Mesa technologies) and motion tracking systems (eg, Vicon and Optotrac). It was preferred to Kinect Sensor V1 because it provides great precision and more stable results [58]. Kinect Sensor V2 is a depth RGB (Red, Green, Blue) image sensor that provides information about the depth, color, and skeleton of a user who is standing in front of the sensor. Therefore, it can track and monitor full-body movements in 3D coordinates (ie, the x-axis, y-axis, and z-axis). It emits a grid of infrared light, and the distance of objects within the camera’s recording range (0.5-4.5 m) is calculated using time-of-flight analysis of reflected light beams, which yields a depth model of the surrounding structures. On the basis of machine learning techniques, the software development kit of Kinect Sensor V2 allows the detection of human shapes and provides an artificial skeleton based on 25 anatomical landmarks (“Kinect joints”) projected into these shapes based on depth data [59].

Kinect Sensor V2 acquired data at a rate of 30 frames per second, and all the tracked body joints were first filtered by using a third-order, low-pass Butterworth filter with a cutoff frequency of 5 Hz. The filter order and bandwidth were optimized to achieve the best results. The angle measures, velocity, and acceleration were computed for each joint type (eg, knee, shoulder, and neck) for each body frame. These measures were used to define the *gesture correctness*, calculated as the percentage of error (<5%) between the expected gestures shown by the avatar of a Pilates teacher and those executed by the user. The teacher’s avatar gesture database was recorded, tagged, and compiled using a visual gesture builder app. On the basis of the gesture correctness, we also defined the reward and feedback system.

The hardware configuration of MS-FIT consists of other 3 devices: a minicomputer containing the exergame (eg, Intel Next Unit Computer), a monitor with a high-definition multimedia interface connector, and a power lead controller to access the exergame and move through the menus. Moreover, a chair without an armrest is needed for some exercises.

The Kinect sensor (in front) and the monitor (at the back) have to be set on a cabinet (or table) at a height of approximately 0.8 m. The user has to stay approximately 2 to 3 m from the Kinect sensor (Multimedia Appendix 2). At the first use of MS-FIT, the user has to calibrate the system, standing with the arms along the body for approximately 5 seconds in front of the sensor. The calibration will be valid for the next sessions also.

#### Phases 3 and 4: Co-Design With MS Health Professional Experts and People With MS

An Italian panel of experts in the field of MS rehabilitation composed of 12 neurologists, 3 physiatrists, 1 statistician, and 1 bioengineer (refer to Multimedia Appendix 3 for a list of participating centers) was invited to participate in the tool co-design [60]. Overall, 2 evaluation cycles were conducted during 2 separate meetings to refine the low-fidelity and high-fidelity prototypes. The evaluation was conducted by using a think-aloud method [61].

During the first cycle, the meeting started with a quick presentation of the aims of phases 1 and 2, followed by a demonstration of the low-fidelity prototype. Key questions regarding the advantages, disadvantages, and potential modifications were asked after each proposed feature. They suggested some preliminary customizations necessary for use with people with MS. Such modifications were mainly related to how the exercises should be implemented to take into account the different levels of physical disabilities of people with MS (eg, adaptation to safe positions).

Similarly, a first cycle of assessment was also conducted on the low-fidelity prototype with 60% (6/10) of people with MS and 40% (4/10) of physical therapists with experience in MS. They were required to specifically focus on user interface interaction, sound effects, and color contrast. Impaired movements and sensory loss (ie, somatosensory, auditory, and visual) such as those experienced by people with MS require specific user interface adaptations. People with MS proposed to use travel around the world as the game’s context instead of a numbered track or an urban map; it was considered to be more motivating for themselves given that, often, MS limits the possibility to travel for both job and tourism.

Feedback from the first cycle was analyzed and used to modify the prototype before the second cycle. Experts, people with MS, and physical therapists agreed on the necessity of an introductory screen that reports safety information; moreover, they agreed on the necessity to wear comfortable clothes (eg, a tracksuit) while using the tool. No other relevant additional modifications were necessary.

### Part 2: Usability Evaluation With Targeted Users

Part 2 was deputed to evaluate the usability of MS-FIT (release 1.0) with people with MS; an ad hoc questionnaire was used to analyze the ease of use, playability, enjoyment, satisfaction, and acceptance.

#### Participants

People with MS were enrolled at the Rehabilitation Service of the Italian Multiple Sclerosis Society in Genoa, IRCCS Institute of the Neurological Sciences of Bologna in Bologna, and Neurological Institute Carlo Besta IRCCS Foundation in Milan. The inclusion criteria were (1) age ≥18 years, (2) all MS courses (relapsing remitting, secondary progressive, and primary progressive), (3) disability level 2 to 4 as determined by the Expanded Disability Status Scale [62], (4) normal cognitive function (determined by a Mini-Mental State Examination score >24) [63], (5) no mood disorders as determined by a Hospital Anxiety and Depression Scale score <10 in the 2 subsets for anxiety and depression [64], (6) ability to maintain balance as determined by a Berg Balance Scale score >46 [65], and (7) at least 1 month without having been treated with rehabilitation. The exclusion criteria were (1) visual deficits that could compromise the use of MS-FIT and (2) relapses in the past 3 months.

#### Protocol

Each participant was involved in 3 outpatient usability test sessions. Before the first session, they received a comprehensive description of the MS-FIT concept and rules; furthermore, they learned to autonomously assemble and calibrate the MS-FIT tool. After the calibration, the participant could play the exergame.

Recent MS recommendations [66] encourage people with MS to practice ≥150 minutes per week of exercise or ≥150 minutes per week of lifestyle physical activity. For this reason, MS-FIT should be routinely used at home for 30 minutes per day for at least 5 days a week. Therefore, a minimum of 30 minutes per session was considered as proof of concept for the usability study.

#### Outcomes

Before the first session, participants were administered a questionnaire investigating their inclination to play traditional games (eg, game of checkers and cards; options: yes or no) and video games and exergames (options: nothing, consoles, tablets/smartphones, and PC), how long they play video games and exergames (options: 0, <4, 4-10, and >10 hours per month), and whether they agree (options: strongly agree, agree, do not know, disagree, and strongly disagree) that a digital assistant is an appealing way to present exercises.

After the last session, participants were asked to rate their experience through an ad hoc, 8-item questionnaire evaluating usability in terms of ease of use, playability, enjoyment, satisfaction, and acceptance, similarly to previous studies [67,68]. All items were rated on a 5-point scale ranging from 1 (strongly disagree) to 5 (strongly agree). Moreover, the usability was determined with a structured interview about the impressions of participants toward MS-FIT features in terms of rules, mechanics, interfaces, and scoring (Table 2).

In addition, the therapists involved in the user test were administered a structured interview about the interaction the participants had with MS-FIT. Specifically, we inquired them about whether the patients were autonomous at the end of the third session (options: yes/no) in using both hardware and software and how long the patients took to set up the system and start to play (options: <5, 5-10, 10-15, 15-20, and >20 minutes). Moreover, a free-text question was administered about their impressions and suggestions regarding the tool in terms of rules, mechanics, interfaces, and scoring.

Feedback from both interviews has been used by the research team to update MS-FIT to a new release for use in MS (MS-FIT 2.0 release).

**Table 2:** Questions of the ad hoc, 8-item questionnaire administered to the participants and the related scores.

Domain and question			Participants’ score																			Score, mean (SD)
			P^a^1		P2		P3		P4		P5		P6		P7		P8		P9			
**Ease of use**																						
	Are the instructions on the MS-FIT^b^ hardware configuration clear to follow?	4		4		3		4		4		4		4		4		4		3.89 (0.33)		
	Is the hardware configuration easy in practice?	4		4		4		4		4		4		4		4		2		3.78 (0.67)		
	Are the instructions on the MS-FIT use clear and easy to follow?	4		4		4		4		5		4		4		4		4		4.11 (0.33)		
	Is the user interface pleasant?	4		4		4		4		4		3		4		4		3		3.78 (0.44)		
**Playability**																						
	Is MS-FIT easy to be played?	5		4		4		4		4		4		4		4		2		3.89 (0.78)		
**Enjoyment**																						
	Does a virtual trainer increase the enjoyment towards exercise?	4		4		4		4		5		3		4		4		5		4.11 (0.60)		
**Satisfaction**																						
	Are you satisfied of the experience with MS-FIT?	3		4		3		4		5		4		4		4		4		3.89 (0.60)		
**Acceptance**																						
	Are you motivated to use MS-FIT autonomously at home?	3		3		3		5		5		5		5		5		5		4.33 (1)		

^a^P: participant.

^b^MS-FIT: Multiple Sclerosis Fitness Intervention Training.

#### Data Analysis

Descriptive statistics were used to describe both participants’ characteristics and their scores on the ad hoc questionnaire. All data were analyzed using STATISTICA 7.1 (StatSoft).

#### Follow-Up

In total, 14 therapists with specific backgrounds in MS (one for each center; Supplementary Material 3) participated in a dedicated meeting in Milan to evaluate the MS-FIT 2.0 release and to suggest eventual further refinements.

## Results

### Part 1: Design and Development of a Pilates Exergame

MS-FIT is a tool for individual, internet-based, game-based training using Microsoft Kinect Sensor V2. The high number of implemented exercises (Multimedia Appendix 1) and the different levels of difficulty prevent boredom.

**Figure 2:**
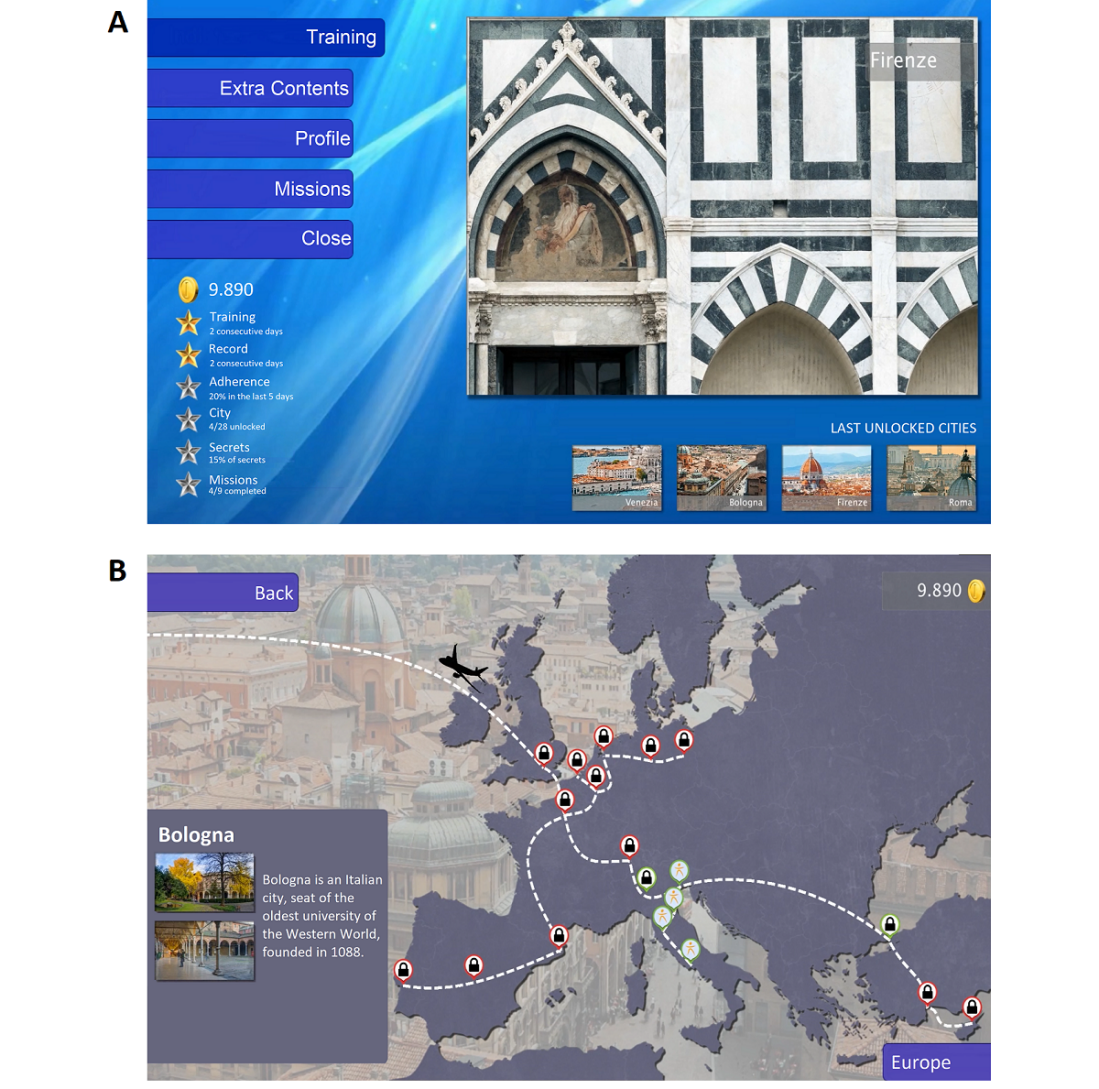
(A) The main interface of Multiple Sclerosis Fitness Intervention Training. On the left, the different menus allow accessing the training, extra contents, user’s profile, and unlocked missions and closing the program; a report of performances and metrics is also present. On the right, a representative picture of the current city and the preview of the last 4 unlocked cities are shown. (B) shows an example of the stays of the travel in Europe. In this case, Rome, Florence, Bologna, and Venice were already unlocked; the user has returned from Venice to Bologna to continue to Greece or Milan.

**Figure 3:**
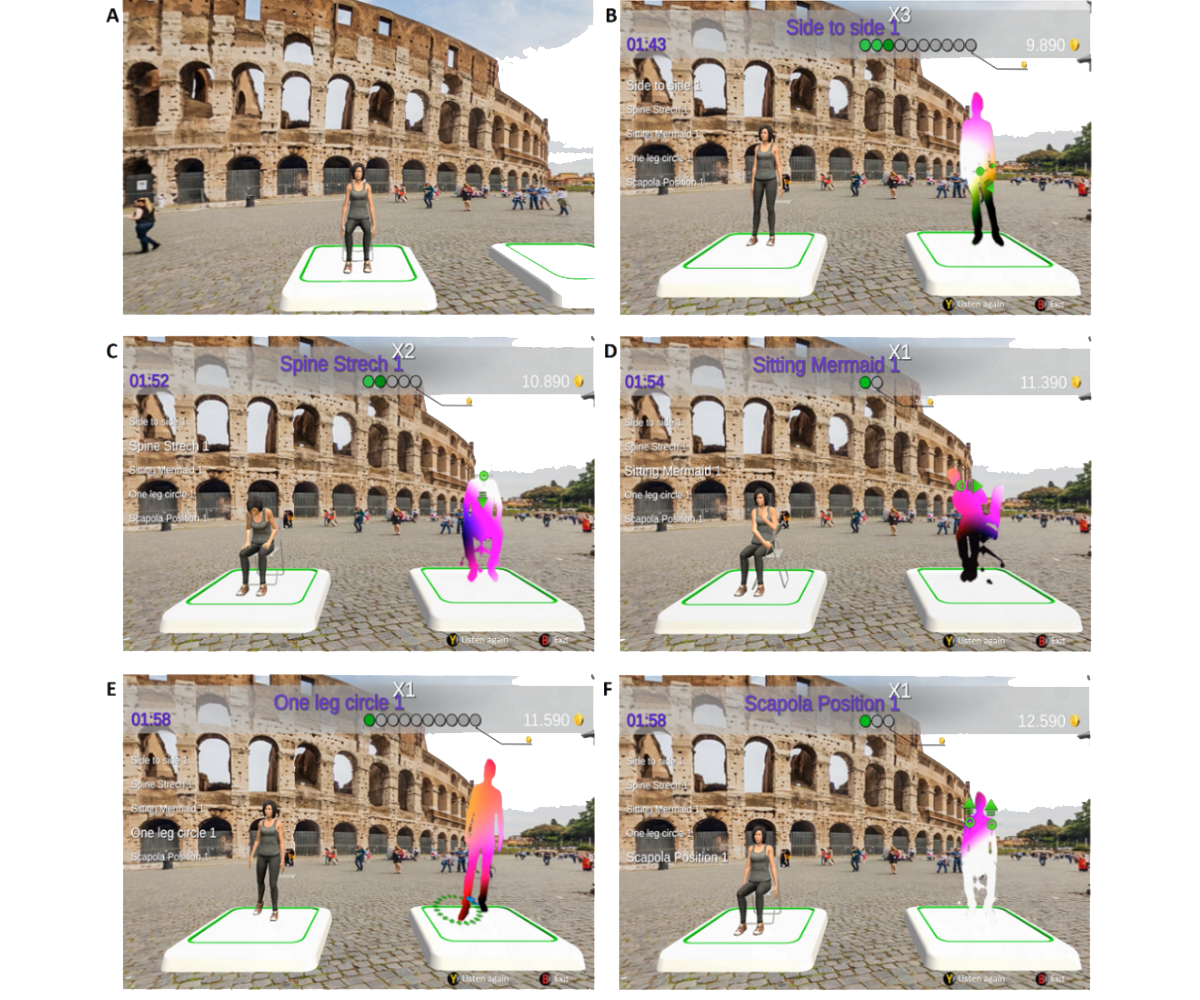
The execution of some exercises implemented in the exergame. During the preview of the exercise (A), the teacher stays in the center of the view; she shows and explains the next exercise that the user will have to perform. Then, she moves to the left, and the user’s avatar is displayed on the right. (B) to (F) represent instants of the execution of Side to side 1, Spide Stretch 1, Sitting Mermaid 1, One leg circle 1, and Scapola Position 1.

The MS-FIT context is *travel* around the world to visit some of the most important cities (Figure 2 and Multimedia Appendix 4) and to learn the aspects of their culture through pictures (eg, landscape) and stories (eg, curiosity; Textbox 1). At each stay of the travel, a teacher of Pilates, represented by an avatar, shows and explains to the user the exercise they will have to perform; a related caption is displayed. The teacher’s avatar (a human model wearing web-based garments) is represented on the left of the screen, whereas the user’s avatar (a human model in a density gradient visualization) is represented on the right and allows real-time feedback to the participant (Figure 3).

Accessibility was guaranteed through the implementation of exercises tailored for people with MS and owing to the use of auditory instructions provided by the teacher’s avatar before starting the exercise while showing the related captions.

The challenge for the user is the execution of the exercises as correctly and accurately as possible at the same time as the teacher; green arrows (or circles for circular gestures), applied to the user’s body joint and segment mainly involved in the exercise, are displayed to suggest the correct direction and to guide the execution (Figure 3). For each correct gesture, the user is rewarded with web-based coins visible while gaming; the reward represents the feedback about performances given to the participant while playing MS-FIT. The accumulated web-based coins can be used to unlock the next stay (ie, next city) and progress along the travel; this progress represents another feedback to the user. Besides this modality of game progress, MS-FIT also provides several missions; for example, twice-daily trainings for 5 consecutive days allow completing the mission, *Methodic* (Multimedia Appendix 5).

As the game progresses, the user develops more skills that motivate them to continue the training; according to the travel progress, the level of difficulty in the next city increases in terms of the number of consecutive repetitions of the same exercise or in terms of exercise complexity; however, the user can also repeat the exercises of previously unlocked cities without training at increasing levels of difficulty.

All these aspects of MS-FIT are expected to be crucial for the engagement of the users and the success of the product.

MS-FIT collects use data and exercise metrics about the frequency and duration of exercise sessions, types of exercises that are performed or skipped by the user, performance in each single exercise, and other statistics (eg, the ratio of unlocked cities to total cities and the ratio of completed missions to total missions). However, these in-game metrics are not shown to the user and were not investigated in this usability study. All data are anonymized and sent to a central server for storage.

**Stories about a city—an example of stories related to Venice. In Multiple Sclerosis Fitness Intervention Training, each story is associated with the corresponding picture.**

**General**

Venice is the capital of the Veneto region. It is built on a group of 118 islands separated by canals and linked by many bridges. During the Middle Ages and Renaissance the city played a central financial and maritime role in Europe. Today Venice is one of the most popular tourist destinations in Europe, so that most of its economy is based on tourism. One of the greatest challenges for Venice in the next future will be to face erosion and rising seas that threaten the delicate balance of this unique city in the world.

**History**

The popular class of Venice was divided into two factions: Castellani and Nicolotti. The former lived in the eastern part, i.e. the industrial area, of ​​the city, while the latter prevalently resided on western area and were mainly engaged in fishing. Venice turned a blind eye to the skirmishes between the two factions, so much to permit them to clash during the so-called “Wars of the Fists.” These battles took place on the bridges of Venice (one of these is still named “Ponte dei Pugni,” i.e. “Bridge of Punches”) and consisted of people fighting each other with bare hands. The fights could be one-on-one, multiple or as battle to take the bridge. During the clashes many contenders fell into the water, often injured, and more rarely someone dead. In 1705 Venice forbade these battles forever, replacing them with the custom of the “Forces of Hercules.”

**Monuments**

The bell tower of of St Mark’s Basilica initially had five bells used for different purposes. The “Maleficio” bell announced an imminent death sentence; the “Marangona” bell marked the beginning and the end of the Arsenale’s workshift; the “Mezzana” bell rang at noon; the “Pregadi” bell announced the start of a meeting at the Doge’s Palace; the “Trottera” bell, which takes its name from the trot of the horses, summoned the nobles to the Doge’s Palace. Of these only the “Marangona” was saved from the collapse of the bell tower of St Mark’s Basilica.

**Curiosity**

In the gardens of the Biennale of Venice there is a statue of Garibaldi on which a funny legend was born. The protagonist of this story is Giuseppe Zolli, a Venetian Garibaldian; it is said he swore to Garibaldi his protection for eternity. In 1921 some witnesses claimed to have seen the ghost of Zolli next to the statue of Garibaldi, intent on persecuting anyone who approached it. The inhabitants erected a statue of Zolli behind that of his protege, to allow him to always watch over him. The ghost has not been seen since.

### Part 2: Usability Evaluation With Targeted Users

#### Participants

A total of 9 people with MS (n=4, 44% women) participated in the usability phase. Their mean age was 42.89 (SD 11.97; range 22-61) years. Of the 9 participants, 8 (89%) were relapsing remitting, and 1 (11%) was secondary progressive; the mean Expanded Disability Status Scale score was 3.17 (SD 0.75; range 2-4), and the mean disease duration was 10.19 (SD 9.18) years.

Of the 9 participants, 6 (67%) did not have experience with video games, 1 (11%) played for <4 hours per month, and 2 (22%) played for 4 to 10 hours per month. Those who played video games used smartphones and tablets. Only 22% (2/9) of the participants reported to be traditional game players. In general, they agreed to consider a digital assistant (an avatar in MS-FIT) as a useful way to make exercise more appealing.

#### User Experience With MS-FIT

All people with MS (9/9, 100%) completed the 3 outpatient sessions of the user test and answered the ad hoc questionnaire. The average score on each item ranged from 3.78 and 4.11, which indicated a high usability level. The 8-item, ad hoc questionnaire scores are illustrated in Table 2.

As reported by the 5 therapists involved in the user test, of the 9 participants, all (n=9, 100%) were autonomous in using both hardware and software at the end of the third session, 6 (67%) needed <10 minutes to set up the system, and 3 (33%) needed 10 to 15 minutes.

At the end of the third session, on average, they visited 2 cities (range 1-3; Rome and Florence) and collected 1044 coins (range 945-1324).

Feedback and suggestions provided by the 9 people with MS and the 5 therapists are presented in Textbox 2 and were implemented in the MS-FIT 2.0 release.

A few final refinements were suggested by the 14 therapists participating in the follow-up meeting and have been implemented in the MS-FIT 2.0 release.

Thus, MS-FIT 2.0 was ready to be tested in a feasibility or a randomized controlled trial (RCT) study.

**Feedback and suggestions provided by people with multiple sclerosis (MS) and therapists.**

**Feedback and suggestions from people with MS**

- Increment of the variety of exercises (especially for standing balance)
- Possibility of standby during the execution of an exercise (not only pause at the end of an exercise)
- Tailoring of treatment plan (eg, based on the disability level)
- Increment of the font size of writings (difficulty in reading for people with MS)
- Necessity of more colloquial terms for the instructions (very technical language is difficult to understand)
- Improvement of the reward system (need for visual feedback besides verbal feedback)

**Feedback and suggestions from therapists**

- Increment of the variety of exercises
- Possibility of detecting an aid (especially in the monopodalic exercises)
- Necessity of more colloquial terms for the instructions (very technical language is difficult to understand)
- Improvement of the reward system (although motivational feedback was implemented, more attention was required for negative performances)

## Discussion

### Principal Findings

In this study, we aimed to design, develop, and user test MS-FIT, an internet-based, game-based tool deputed to deliver Pilates exercises for people with MS. Study outcomes support the therapeutic use of Pilates in MS management because it is a safe, active treatment method (few adverse effects) with high adherence (low dropout rate) and can improve important parameters in the target population, such as balance, gait, physical functional capacities, and even cognitive functions [69].

MS-FIT was formulated by integrating the principal knowledge and existing evidence from MS literature and by subjecting the exergame to critical appraisal from patients and experts. We adopted a research-based and iterative development process that considered the knowledge gained from different disciplines (human movement science, rehabilitation, physical activity, game design, and research) and users’ perspectives (people with MS and therapists) to holistically generate a potentially attractive and effective user-centered exergame for people with MS.

Although the participants had few experiences with exergames/video games and consoles, they became autonomous in using MS-FIT after only 3 sessions. All of them (9/9, 100%) completed the three 30-minute sessions and reported finding the tool easy to use, playable, enjoyable, and satisfactory. This result was corroborated by the quantity of coins gained and the number of cities unlocked, which could be considered as a surrogate index of the ease to use, playability, and acceptance. Feedback and suggestions provided by people with MS and therapists allowed for increasing the number of implemented exercises and, overall, improving the quality of the MS-FIT exergame.

### Comparison With Previous Studies

In MS-FIT, we used Microsoft Kinect because it has been reported to be well accepted by users and for its effectiveness in several rehabilitative interventions for people with MS. For example, RemoviEM [36] is an intuitive and motivating way to train weight transfer and upper limb coordination of people with MS and to improve balance and mobility. Similarly, Molhemi et al [37] demonstrated the efficacy of a Kinect-based rehabilitative tool to train standing, walking, and weight shifting in MS. In addition, more consolidated experiences with a portable, low-cost tool such as the Nintendo Wii Balance Board could pave the way for an easy future adoption of Kinect-based exergames into clinical practice [24]. In this context, Brichetto et al [70] investigated the effectiveness of balance training based on the Nintendo Wii Balance Board versus a traditional rehabilitative training. Despite the excellent compliance with both interventions, a high clinically significant improvement in balance was shown in the group using the exergame. In addition, Prosperini et al [71] showed that training with the Nintendo Wii Balance Board induced microstructure modifications of the superior cerebellar peduncles, suggesting that high-intensity and task-oriented exercises such as those implemented in the Nintendo Wii Fit Plus package could favor brain reorganization in people with MS. Overall, internet-based, game-based training is an effective and efficient approach for empowering user engagement, which contributes to positive outcomes of an intervention [72,73].

To limit high dropout rates owing to potential low engagement with modern digital technology, the UCD approach, which incorporates game design principles (ie, goals, rules, feedback, points, time, reward structures, levels, and esthetics), was used in the process of designing and developing MS-FIT. Many studies demonstrated the benefits of using the UCD concept for developing exergames. For example, Lange et al [18] used the UCD approach to develop an internet-based, game-based program aimed at training the dynamic balance of individuals who had experienced a stroke. Hemingway et al [43] used the UCD process to develop a mobile game to influence the behavior of HIV service uptake among a key population. Howes et al [74] also used UCD to develop a bespoke exergame to deliver strength and balance exercise programs to older adults. Similarly, Kamnardsiri et al [34] developed and tested an internet-based, physical-cognitive, game-based training system using Kinect for older individuals. Therefore, the UCD approach is a core process that should be embedded in health game development to ensure usability and acceptability of and full benefit from exergame technology.

The findings from this study seem to be confirming these results. Consistent with previous studies, our game system provides feedback, including scores and performance outcomes, which enhances the motivation of the users [75,76]. However, it was not possible to evaluate the clinical benefit of MS-FIT because the number of user test sessions was very low; future planned studies will assess this aspect.

To the best of our knowledge, MS-FIT is the first exergame designed and developed to deliver Pilates exercises through an internet-based, game-based technology. Although MS-FIT was customized for people with MS, few adaptations would be necessary for use in the context of other clinical conditions [77-80].

### Plans for Future Studies

The design, development, and usability testing of MS-FIT is the first step of a large research plan including an ongoing feasibility study followed by an RCT. The feasibility study has been planned to assess the satisfaction and acceptance toward the tool, adherence to the intervention, and dropout rate on a large sample size and for a long period of more intense use (ie, 5 sessions a week for 6 weeks). The use of validated questionnaires investigating usability [81-83] and the identification of potential bugs will be useful for further tool refinement. Moreover, the administration of clinical tests evaluating balance (eg, timed up-and-go test) will allow for estimating the effect of the intervention and its variance, which are necessary to calculate the appropriate sample size for the RCT. Finally, the feasibility study will allow the identification of groups of exercises with a level of difficulty that is suitable for the specific needs and capabilities of people with MS in different clinical stages.

An RCT (ClinicalTrials.gov; ID NCT04011579) will assess the effect of MS-FIT on physical and cognitive domains, mood, and QoL of people with MS. If RCT results are positive, MS-FIT could be proposed in combination with rehabilitation to potentiate the rehabilitative intervention effect or guarantee the continuity of care of people with MS.

Owing to the discontinuation of the Kinect Sensor V2, for the continued development of MS-FIT and successful translation into clinical practice, a new compatible commercial sensor must be identified and the app should be adapted. The Microsoft AzureKinect demonstrated sufficiently high interrater and intrarater reliability when compared with the Kinect Sensor V2. Moreover, a full-body skeletal tracking library in Python has recently become available. These aspects suggest that the Microsoft AzureKinect may be an adequate replacement within the MS-FIT system [84]. Another alternative solution to the Kinect Sensor V2 is MentorAge, a depth RGB (Red, Green, Blue) image sensor tracking system that operates on the Android system and uses infrared 3D capturing technology. It can detect a maximum of 4 people in a single room within a range of 0.6 to 5 m and has proven its capabilities and potentialities in real-life scenarios [80,85,86]. No additional equipment is thought to be used compared with the current version of MS-FIT.

### Limitations

Some limitations of the study need to be acknowledged. First, the evaluation of the target user’s experience involved a small number of participants. Furthermore, the usability results were obtained from a few training sessions. Thus, the findings should be considered as preliminary and interpreted with caution. Second, the minimum requested distance between the Kinect sensor and user could limit at-home use. New versions of the Kinect sensors could allow overcoming this limitation. Finally, this study investigated the usability by using an ad hoc questionnaire; however, in a future feasibility study, the use of validated questionnaires should be encouraged.

### Conclusions

MS-FIT is an exergame using the Microsoft Kinect sensor for outpatient and at-home MS balance interventions. It digitally implements Pilates exercises for breathing, posture, and balance. MS-FIT is usable and well accepted by the target users, with positive feedback. Thus, the exergame is a promising tool for enhancing the motivation among people with MS to engage in exercise with the aim of improving their physical status.

## Appendix

Multimedia Appendix 1 Pilates exercises implemented in Multiple Sclerosis Fitness Intervention Training.

Multimedia Appendix 2 Additional illustration of the device and implementation.

Multimedia Appendix 3 List of participating centres.

Multimedia Appendix 4 List of cities.

Multimedia Appendix 5 List of the missions.

## Abbreviations

MS: multiple sclerosis
MS-FIT: Multiple Sclerosis Fitness Intervention Training
QoL: quality of life
RCT: randomized controlled trial
UCD: user-centered design

## Data Availability

The data sets generated and analyzed during this study are available from the corresponding author upon reasonable request.
