# Supplementary material for: An Internet- and Kinect-Based Multiple Sclerosis Fitness Intervention Training With Pilates Exercises: Development and Usability Study
Source: JMIR Serious Games. 2023 Nov 8;11:e41371. doi: 10.2196/41371 (PMC10666018; doi:10.2196/41371)
Supplement: Multimedia Appendix 1 [file games_v11i1e41371_app1.docx]

**Supplementary Material 1. Pilates exercises Implemented in MS-FIT**

**BREATHING**

***Pure breathing***

***Primary aims***: perception of abdominal-costal-sternal breathing, perception of posterior costal breathing, increased respiratory capacity, chest mobilization.

***Secondary aim***: axial alignment and stabilization of the backbone in neutral position and in flexion while seated during the respiratory cycle.

***Position***: seated on a stool, backbone in neutral position, feet firmly on the ground and aligned with knees and hips, arms at sides.

***Exercises***:

- ***Abdominal breathing***
  - *Instructions*. Place one hand on the abdomen below the navel. Keep the backbone stabilized and breathe in gradually controlling the movement and counting up to 3. Breathe out gradually controlling the movement and counting up to 5. Repeat 2/4 times.
  - *Imagery*. Imagine using breathed in/breathed out air to inflate/deflate a balloon placed inside the abdomen.
- ***Sternal breathing***
  - *Instructions*. Place one hand on the sternum. Keep the backbone stabilized and breathe in gradually lifting the sternum upwards; control the movement counting up to 3. Breathe out and let the sternum gradually returns to the starting position, controlling the movement and counting up to 4. Repeat 2/4 times.
  - *Imagery*. Imagine a wire attached to the sternum that lifts it upwards and takes it back downwards every inhalation/exhalation.
- ***Costal breathing***
  - *Instructions*. Place the hands laterally on the chest at the level of low ribs. Keep the backbone stabilized, breathe in and gradually widen the ribs laterally against the hands, controlling the movement and counting up to 3. Breathe out and gradually move the ribs away from the hands, controlling the movement and counting up to 5. Repeat 2/4 times.
  - *Imagery*. Imagine the chest as a fireplace plunger pointing upwards: extend the plunger to take in air while breathing in and empty the plunger while breathing out.
- ***Posterior costal breathing***
  - *Instructions*. Lean forward with the torso and place the hands postero-laterally on the chest. Keep the backbone stabilized during the flexion (without cowering), breathe in and gradually widen the ribs laterally and posteriorly against the hands; control the movement and counting up to 3. Breathe out and gradually move the ribs away from the hands, controlling the movement and counting up to 5. Repeat 2/4 times.
  - *Imagery*. Imagine using breathed in/breathed out air to inflate/deflate two balloons placed on the backbone sides at the level of low ribs.

***Sitting Mermaid***

***Primary aims***: perception of lateral-costal breathing, increased respiratory capacity, chest mobilization.

***Secondary aims***: stretching and lateral hold of the trunk while lengthening in lateral flexion, lateral stabilization of the pelvis, mobilization in abduction-adduction of the scapula-humeral joint, dynamic stabilization of the shoulder girdle during the lateral flexion of the trunk, Core activation while breathing, correct trunk-head alignment during lateral flexion.

***Position***: seated on a chair with the backrest on a side, backbone in neutral position, feet firmly on the ground and aligned with knees and hips.

***Exercises***:

- ***Mermaid arm to chest 1***
  - *Instructions*. Bring the forearm (opposite the backrest) in front of the abdomen and grasp the backrest of the chair with the hand. Place the other arm on the chest. Slightly contract pelvic floor and transversus abdominis keeping them activated throughout the exercise. Breathe in while axially lengthening the backbone in neutral position. Breathe out by laterally leaning the torso opposite the backrest without detaching the buttocks from the seat. Keep the buttocks leaning on the seat by pulling yourself towards the backrest with the grasping hand throughout the exercise. Breathe in returning to the starting position. Repeat at least 3 times on each side.
  - *Imagery*. Imagine leaning laterally to step over a stick at the side of the body without touching it.
- ***Mermaid arm to chest 2***
  - *Instructions*. Bring the forearm (opposite the backrest) in front of the abdomen and grasp the backrest of the chair with the hand. Place the other arm on the chest. Slightly contract pelvic floor and transversus abdominis keeping them activated throughout the exercise. Breathe in while axially lengthening the backbone in neutral position. Breathe out by laterally leaning the torso opposite the backrest without detaching the buttocks from the seat. Keep the torso stabilized during the lateral flexion; breathe in and breathe out 2/3 times progressively increasing the expansion and closing ribs movements. Finally, return to the starting position. Keep the buttocks leaning on the seat by pulling yourself towards the backrest with the grasping hand throughout the exercise. Breathe in returning to the starting position. Repeat at least 3 times on each side.
  - *Imagery*. Imagine leaning laterally to step over a stick at the side of the body without touching it.
- ***Mermaid arm over the head 1***
  - *Instructions*. : Bring the forearm (opposite the backrest) in front of the abdomen and grasp the backrest of the chair with the hand. Slightly contract pelvic floor and transversus abdominis keeping them activated throughout the exercise. Abduct and lift the other arm over the head without elevating the shoulder. Breathe in while axially lengthening the backbone in neutral position. Keep the shoulder girdle stabilized and the arm lifted; breathe out by laterally leaning the torso opposite the backrest without detaching the buttocks from the seat. Breathe out returning to the starting position. Keep the buttocks leaning on the seat by pulling yourself towards the backrest with the grasping hand throughout the exercise. Repeat at least 3 times on each side.
  - *Imagery*. Imagine leaning laterally to step over a stick at the side of the body without touching it.
- ***Mermaid arm over the head 2***
  - *Instructions*. : Bring the forearm (opposite the backrest) in front of the abdomen and grasp the backrest of the chair with the hand. Slightly contract pelvic floor and transversus abdominis keeping them activated throughout the exercise. Abduct and lift the other arm over the head without elevating the shoulder. Breathe in while axially lengthening the backbone in neutral position. Keep the shoulder girdle stabilized and the arm lifted; breathe out by laterally leaning the torso opposite the backrest without detaching the buttocks from the seat. Keep the torso stabilized during the lateral flexion; breathe in and breathe out 3 times progressively increasing the expansion and closing ribs movements. Finally, return to the starting position. Keep the buttocks leaning on the seat by pulling yourself towards the backrest with the grasping hand throughout the exercise. Repeat at least 3 times on each side.
  - *Imagery*. Imagine leaning laterally to step over a stick at the side of the body without touching it.
- ***Mermaid with active stabilization 1***
  - *Instructions*. Bring the hand (of the arm opposite the backrest) along the side. Slightly contract pelvic floor and transversus abdominis keeping them activated throughout the exercise. Abduct and lift the other arm over the head without elevating the shoulder. Breathe while axially lengthening the backbone in neutral position. Keep the shoulder girdle stabilized and the arm lifted; breathe out by laterally leaning the torso opposite the backrest without detaching the buttocks from the seat. Breathe in returning to the starting position. Repeat at least 3 times on each side.
  - *Imagery*. Imagine leaning laterally to step over a stick at the side of the body without touching it.
- ***Mermaid with active stabilization 2***
  - *Instructions*. Bring the hand (of the arm opposite the backrest) along the side. Slightly contract pelvic floor and transversus abdominis keeping them activated throughout the exercise. Abduct and lift the other arm over the head without elevating the shoulder. Breathe while axially lengthening the backbone in neutral position. Keep the shoulder girdle stabilized and the arm lifted; breathe out by laterally leaning the torso opposite the backrest without detaching the buttocks from the seat. Keep the torso stabilized during the lateral flexion; breathe in and breathe out 3 times progressively increasing the expansion and closing ribs movements. Finally, return to the starting position. Breathe in returning to the starting position. Repeat at least 3 times on each side.
  - *Imagery*. Imagine leaning laterally to step over a stick at the side of the body without touching it.

***Kneeling Mermaid***

***Primary aims***: perception of lateral-costal breathing, increased respiratory capacity, chest mobilization.

***Secondary aims***: stretching and lateral hold of the trunk while lengthening in lateral flexion, lateral stabilization of the pelvis, mobilization in abduction-adduction of the scapula-humeral joint, dynamic stabilization of the shoulder girdle during the lateral flexion of the trunk, Core activation while breathing, correct trunk-head alignment during lateral flexion.

***Position***: kneeling, abduct the arms horizontally towards the shoulder on the scapular plane. Left palm facing up and right palm facing down. Slightly contract pelvic floor and transversus abdominis keeping them activated throughout the exercise.

***Exercises***:

- ***Kneeling Mermaid 1***
  - *Instructions*. Breathe in while axially lengthening the backbone in neutral position. Keep the shoulder girdle stabilized and breathe out by leaning the torso on the right; elevate the left arm over the head and lower the right arm towards the ground. Do not move the pelvis laterally or backwards while leaning the torso: the pelvis must stay put and the legs parallel to each other. Breathe in returning to the starting position. Repeat at least 3 times on each side.
- ***Kneeling Mermaid 2***
  - *Instructions*. Breathe in while axially lengthening the backbone in neutral position. Keep the shoulder girdle stabilized and breathe out by leaning the torso on the right; elevate the left arm over the head and lower the right arm towards the ground. Do not move the pelvis laterally or backwards while leaning the torso: the pelvis must stay put and the legs parallel to each other. Keep the torso stabilized during the lateral flexion; breathe in and breathe out 3 times progressively increasing the expansion and closing of the ribs movements. Finally, return to the starting position. Breathe in returning to the starting position. Repeat at least 3 times on each side.

***Sitting arms circles***

***Primary aims***: increased respiratory capacity, chest mobilization.

***Secondary aims***: mobilization in flexion-extension and in circumduction of the scapula-humeral joint, coordination breathing-movement, axial backbone self-lengthening and backbone stabilization in neutral position while seated, Core activation integrated with breathing and upper limbs movement, correct trunk-head alignment during arm movements.

***Position***: seated on a stool, backbone in neutral position, feet firmly on the ground and aligned with knees and hips.

***Exercises***:

- ***V position prep***
  - *Instructions*. Slightly contract pelvic floor and transversus abdominis keeping them activated throughout the exercise. Keep the backbone stabilized and breathe in while elevating the arms over the head in "V" position and counting up to 4; breathe out while lowering the arms and counting up to 5. Breathe in, lift the sternum, widen the ribs and slightly inflate the belly trying to distance chest and pelvis (without extending the backbone). Breathe out, release sternum and ribs, and slightly push the belly in, maintaining the distance gained between chest and pelvis. Repeat at least 3 times.
  - *Imagery*. Imagine a wire attached to the top of the head that lifts you higher and higher.
- ***Sitting arm circles***
  - *Instructions*. Slightly contract pelvic floor and transversus abdominis keeping them activated throughout the exercise. Keep backbone and shoulder girdle stabilized, draw circles with the arms; breathe in while elevating the arms over the head and slowly counting up to 4; breathe out while lowering the arms and counting up to 5. Breathe in, lift the sternum, widen the ribs and slightly inflate the belly trying to distance chest and pelvis (without extending the backbone). Breathe out, release the sternum and ribs, and slightly push the belly in (without flexing the backbone), maintaining the distance gained between chest and pelvis. Repeat at least 4 times.
  - *Imagery*. Imagine a wire attached to the top of the head that lifts you higher and higher.
- ***Sitting arm circles (speed)***
  - *Instructions*. Slightly contract pelvic floor and transversus abdominis keeping them activated throughout the exercise. Keep backbone and shoulder girdle stabilized, draw circles with the arms; breathe in while elevating the arms over the head and breathe out while lowering the arms with a sustained rhythm 3 times; then, change the rotation direction. Keep backbone stabilized and chest lifted upwards during the movements with the arms (without extending the backbone). Repeat at least 2/4 times.
  - *Imagery*. Imagine a wire attached to the top of the head that lifts you higher and higher.
- ***Sitting arm circles (speed + endurance)***
  - *Instructions*. Slightly contract pelvic floor and transversus abdominis keeping them activated throughout the exercise. Keep backbone and shoulder girdle stabilized, draw circles with the arms; breathe in while elevating the arms over the head and breathe out while lowering the arms with a sustained rhythm 5 times; then, change the rotation direction. Keep backbone stabilized and chest lifted upwards during the movements with the arms (without extending the backbone). Repeat at least 4 times.
  - *Imagery*. Imagine a wire attached to the top of the head that lifts you higher and higher.

***Standing arms circles***

***Primary aims***: increased respiratory capacity, chest mobilization.

***Secondary aims***: mobilization in flexion-extension and in circumduction of the scapula-humeral joint, coordination breathing-movement, axial backbone self-lengthening and backbone stabilization in neutral standing position, Core activation integrated with breathing and upper limbs movement, correct trunk-head alignment during arm movements.

***Position***: standing, backbone in neutral position, feet aligned.

***Exercise***:

- ***Standing arm circles***
  - *Instructions*. Slightly contract pelvic floor and transversus abdominis keeping them activated throughout the exercise. Keep backbone and shoulder girdle stabilized, draw circles with the arms; breathe in by elevating the arms a little over the eyes and counting up to 4; breathe out while lowering the arms and counting up to 5. Breathe in, lift the sternum, widen the ribs and slightly inflate the belly trying to distance chest and pelvis (without extending the backbone). Breathe out, release the sternum and ribs, and slightly push the belly in (without flexing the backbone), maintaining the distance gained between chest and pelvis. Repeat at least 2/4 times.
  - *Imagery*. Imagine a wire attached to the top of the head that lifts you higher and higher.
- ***Standing arm circles (speed + coordination)***
  - *Instructions*. Slightly contract pelvic floor and transversus abdominis keeping them activated throughout the exercise. Extend the arms parallel forward at the shoulders level with the palms facing down. Keep backbone and shoulder girdle stabilized, draw 3 small circles fastly, firstly in one direction and, after rotating the palms upwards, in the other direction. Breathe in by elevating the arms and breathe out by lowering them. Repeat at least 4 times.
  - *Imagery*. Imagine a wire attached to the top of the head that lifts you higher and higher.
- ***Standing arm circles (speed + coordination + endurance)***
  - *Instructions*. Slightly contract pelvic floor and transversus abdominis keeping them activated throughout the exercise. Extend the arms parallel forward at the shoulders level with the palms facing down. Keep backbone and shoulder girdle stabilized, draw 5 small circles fastly, firstly in one direction and, after rotating the palms upwards, in the other direction. Then, abduct the arms horizontally and keeping the palms upwards draw 5 small circles fastly, firstly in one direction and after rotating the palms downwards, in the other direction. Finally, relax the arms along the sides. Breathe in by elevating the arms and breathe out by lowering them. Repeat at least 4 times.
  - *Imagery*. Imagine a wire attached to the top of the head that lifts you higher and higher.

**POSTURE**

***Arch & Curl***

***Primary aims***: perception of the correct pelvis, chest, head, and shoulder girdle alignment in neutral position while seated; correct trunk-head alignment during flexion-extension, dynamic shoulder girdle stabilization.

***Secondary aims***: mobilization in flexion-extension of backbone, Core activation integrated with breathing and trunk movement.

***Position***: seated on a stool, backbone in neutral position, feet firmly on the ground and aligned with knees hips, hands on thighs, perception of weight on the ischiums.

***Exercise***:

- ***Arch & Curl***
  - *Instructions*. Slightly contract pelvic floor and transversus abdominis keeping them activated throughout the exercise. Breathe in while rotating the pelvis forward by bringing the weight in front of the ischium; chest and head extend following the movement. Breathe out while rotating the pelvis backwards by bringing the weight behind the ischium; chest and head flex following the movement. Repeat at least 3/5 times, and then stop on the ischium and perceive the neutral position of pelvis, chest, head, and shoulder girdle and the backbone alignment.
  - *Imagery*. Imagine moving like an underwater plant that swings sinuously following the wave rhythm.
- ***Arch & Curl with arms***
  - *Instructions*. Slightly contract pelvic floor and transversus abdominis keeping them activated throughout the exercise. Breathe in while sliding the hands on the hips, rotate the pelvis forward by bringing the weight in front of the ischium; chest and head extend following the movement. Breathe out while sliding the hands towards the knees, rotate the pelvis backwards by bringing the weight behind the ischium; chest and head flex following the movement. Repeat at least 3/5 times, and then stop on the ischium and perceive the neutral position of pelvis, chest, head, and shoulder girdle and the backbone alignment.
  - *Imagery*. Imagine moving like an underwater plant that swings sinuously following the wave rhythm.

***Scapula position***

***Primary aim***: perception of the correct position of shoulder girdle while seated with backbone in neutral position.

***Secondary aims***: mobilization of the scapula-thoracic joint, stabilization of backbone in neutral position while seated.

***Position***: seated on a stool, backbone in neutral position, feet firmly on the ground and aligned with knees and hips, arms at sides.

***Exercise***:

- ***Elevation & depression***
  - *Instructions*. Breathing in while elevating the shoulders and breathe out while lowering them. Perform the movements at the maximum possible range. Repeat at least 3 times, and then stop in neutral position and perceive the difference with respect to the other two.
- ***Adduction & Abduction***
  - *Instructions*. Elevate the arms forward at the shoulder level with palms facing each other. Breathe in and stretch the arms forward by sliding the scapula across the chest; breathe out while bringing the arms back by adducting the scapula to the backbone. Repeat at least 3 times, and then stop in neutral position and perceive the difference with respect to the other two.
  - *Imagery*. Imagine the scapula like two bars of soap sliding across the chest.

***Spine stretch***

***Primary aims***: perception of the correct backbone alignment while seated, correct trunk-head alignment during flexion, dynamic shoulder girdle stabilization during the trunk flexion.

***Secondary aims***: axial backbone self-lengthening, lumbar region decompression, dorsal backbone mobilization during flexion, Core activation integrated with breathing and movement in flexion.

***Position***: seated on a stool, backbone in neutral position, legs aligned with hips, knees flexed, feet on the ground.

***Exercise***:

- ***Spine stretch 1***
  - *Instructions.* Arms relaxed at sides with hands on the thighs. Slightly contract pelvic floor and transversus abdominis keeping them activated throughout the exercise. Breathe in while axially lengthening the backbone; breathe out while flexing forward by keeping the pelvis perpendicular to the ground. Breathe in while returning to the starting position by realigning the backbone.
  - *Imagery*. Imagine leaning over a windowsill to observe what is happening in the street.
- ***Spine stretch 2***
  - *Instructions*. Arms elevated forward at the shoulder level with palms facing each other. Slightly contract pelvic floor and transversus abdominis keeping them activated throughout the exercise. Breathe in while axially lengthening the backbone; breathe out while flexing forward by keeping the pelvis perpendicular to the ground. Breathe in while returning to the starting position by realigning the backbone.
  - *Imagery*. Imagine leaning over a windowsill to observe what is happening in the street.

***Spine twist***

***Primary aims***: axial backbone self-lengthening and backbone stabilization while seated, correct trunk-head alignment during rotation, release of the shoulder girdle and correct trunk and upper limbs positioning as preparation to the gait pattern, dynamic stabilization of the shoulder girdle during trunk rotation.

***Secondary aims***: backbone mobilization in rotation with lumbar decompression, Core activation integrated with breathing and rotation.

***Position***: seated on a stool, backbone in neutral position, legs aligned at hips, knees flexed, feet on the ground.

***Exercise***:

- ***Spine twist***
  - *Instructions*. Arms relaxed at sides. Slightly contract pelvic floor and transversus abdominis keeping them activated throughout the exercise. Breathe in while axially lengthening the backbone; breathe out while rotating the chest on the right as far as possible keeping pelvis and shoulder girdle stabilized. Breathe in while returning to the starting position by realigning the backbone. Repeat at least 3 times.
  - *Imagery*. Imagine climbing higher and higher on a spiral staircase.
- ***Spine twist with arms***
  - *Instructions*. Arms elevated forward at the shoulder level with palms facing each other. Slightly contract pelvic floor and transversus abdominis keeping them activated throughout the exercise. Breathe in while axially lengthening the backbone; breathe out while rotating the chest on the right as far as possible while keeping pelvis and shoulder girdle stabilized. Breathe in while returning to the starting position by realigning the backbone. Repeat at least 3 times.
  - *Imagery*. Imagine climbing higher and higher on a spiral staircase.

***Thigh stretch***

***Primary aims***: axial backbone self-lengthening, trunk-hips-knees stabilization in correct alignment during knees flexion-extension, hip flexor muscles stretching, shoulder girdle stabilization.

***Secondary aims***: Core activation integrated with breathing and knees flexion-extension.

***Position***: kneeling with knees aligned with hips; hands forward at the shoulder level with palms facing each other.

***Exercise***:

- ***Thigh stretch***
  - *Instructions*. Slightly contract pelvic floor and transversus abdominis keeping them activated throughout the exercise. Stabilize the backbone in neutral position and axially self-stretch while breathing in. Breathe out while flexing the knees to lean backwards slightly keeping trunk and hips aligned. Breathe in while returning to the starting position.
  - *Imagery*. Imagine taking a "Z" shape with the body.
- ***Maintained thigh stretch***
  - *Instructions*. Slightly contract pelvic floor and transversus abdominis keeping them activated throughout the exercise. Stabilize the backbone in neutral position and axially self-stretch while breathing in. Breathe out while flexing the knees to lean backwards as far as possible keeping trunk and hips aligned. Breathe in and breathe out while maintaining the position, then breathe in and breathe out while returning to the starting position.
  - *Imagery*. Imagine taking a "Z" shape with the body.

***Awareness of correct posture in standing position***

***Primary aims***: perception of the difference between usual and correct posture, postural self-correction.

***Secondary aims***: Core activation integrated with postural self-correction.

***Position***: standing.

***Exercise***:

- ***Awareness of correct posture in standing position***
  - *Instructions*. Correct the posture while adhering to the silhouette, and then return to the usual posture. Perceive the body position changes while returning to the usual posture. Maintain for 3 respiratory cycles while activating pelvic floor and transversus abdominis throughout the exercise.
  - *Imagery*. Imagine adhering to the silhouette.

***Pelvis alignment***

***Primary aims***: pelvis alignment control and maintenance on the frontal plane in standing position and on the side, correct trunk-head alignment in monopodalic stance.

***Secondary aims***: Core activation integrated with breathing and posture maintenance in monopodalic stance: pelvis alignment with balance control.

***Position***: standing, backbone in neutral position, feet firmly on the ground and aligned with knees and hips, arms at sides.

***Exercise***:

- ***Pelvis alignment 1***
  - *Instructions*. Contract pelvic floor and transversus abdominis keeping them activated throughout the exercise. While keeping the pelvis stabilized in alignment, breathe in and lift one foot off the ground by slightly flexing the knee to remain in monopodalic stance. While keeping the pelvis aligned, breathe out and slightly flex the knee of the foot on the ground by remaining in monopodalic stance. Breathe in while extending the knee of the foot on the ground by remaining in monopodalic stance. Repeat at least 4 times on each side.
- ***Pelvis alignment 2***
  - *Instructions*. Contract pelvic floor and transversus abdominis keeping them activated throughout the exercise. While keeping the pelvis stabilized in alignment, breathe in and lift one foot off the ground by slightly flexing the knee to remain in monopodalic stance. Breathe in while lifting upwards the side of the leg suspended from the ground without tilting the torso sideways. Breathe out and bring the pelvis aligned by controlling the relaxation movement. Breathe in, and then breathe out while lowering the side of the suspended leg by controlling the release movement. Breathe in while bringing backwards the pelvis aligned. Repeat at least 3 times on each side.
- ***Side pelvis alignment 1***
  - *Instructions*. Contract pelvic floor and transversus abdominis keeping them activated throughout the exercise. Lie on a side resting on the forearm aligned under the shoulder with the palm of the hand on the floor and the hips and knees flexed. Breathe in while lifting the pelvis off the ground. Breathe out while bringing the pelvis back to the ground by controlling the relaxation movement. Repeat at least 3 times on each side.
- ***Side pelvis alignment 2***
  - *Instructions*. Contract pelvic floor and transversus abdominis keeping them activated throughout the exercise. Lie on a side resting on the forearm aligned under the shoulder with the palm of the hand on the floor and the hips and knees flexed. Breathe in while lifting the pelvis off the ground. Perform 2 respiratory cycles keeping the pelvis lifted, and then bring the pelvis back to the ground while breathing out by controlling the relaxation movement. Repeat at least 3 times on each side.

***Elephant stretch***

***Primary aims***: posterior chain stretching, dynamic hamstrings stretching.

***Secondary aims***: backbone stabilization in alignment during knees flexion-extension.

***Position***: standing.

***Exercise***:

- ***Elephant stretch 1***
  - *Instructions*. Flex the knees and bring palms to the seat. Flex the elbows while leaning the torso downward; flex the hips with the back flatten as much as possible. Breathe in, and then breathe out while extending the knees without changing the back position. Breathe in and flex again the knees just enough to release tension from the hamstrings. Repeat at least 3 times, and then return standing while breathing out.
  - *Imagery*. Imagine a wire attached to the sacrum pulled towards the ceiling while extending the knees.
- ***Elephant stretch 2***
  - *Instructions*. Flex the knees and bring forearms to the seat while leaning the torso downwards; flex the hips with the back flatten as much as possible. Breathe in, and then breathe out while extending the knees without changing the back position. Breathe in and flex again the knees just enough to release tension from the hamstrings. Repeat at least 3 times, and then return standing while breathing out.
  - *Imagery*. Imagine a wire attached to the sacrum pulled towards the ceiling while extending the knees.
- ***Elephant stretch 3***
  - *Instructions*. Flex the knees and bring hands on the ground while leaning the torso downwards; flex the hips with the back flatten as much as possible. Breathe in, and then breathe out while extending the knees without changing the back position. Breathe in and flex again the knees just enough to release tension from the hamstrings. Repeat at least 3 times, and then return standing while breathing out.
  - *Imagery*. Imagine a wire attached to the sacrum pulled towards the ceiling while extending the knees.

**BALANCE**

***Side to side***

***Primary aims***: correct load transfer, monopodalic stance, balance maintenance in monopodalic stance.

***Secondary aims***: Core activation maintained during limb-to-limb load transfer in monopodalic stance, trunk-head alignment maintenance during limb-to-limb load transfer in monopodalic stance, coordination between breathing and movement, scapula-humeral joint mobilization in abduction-adduction.

***Position***: standing, backbone in neutral position, feet firmly on the ground and aligned with knees hips, hands on hips.

***Exercise***:

- ***Side to side***
  - *Instructions*. Contract pelvic floor and transversus abdominis keeping them activated throughout the exercise. While maintaining trunk and pelvis aligned, follow the rhythm; breathe in while shifting the body weight on the right foot, and breathe out while shifting the body weight on the left foot. Repeat at least 5 times (gradually increasing the rhythm).
  - *Imagery*. Imagine being on two stones in a river and having to move alternately on the left and right to escape the currents.
- ***Side to side one feet***
  - *Instructions*. Contract pelvic floor and transversus abdominis keeping them activated throughout the exercise. Maintain trunk and pelvis aligned. Breathe out while shifting the body weight on the right foot and lift the left toe from the ground; breathing in while returning to the starting position. Breathing out while shifting the body weight on the left foot and lift the right toe from the ground. Repeat at least 4 times (gradually increasing the rhythm).
  - *Imagery*. Imagine being on two stones in a river and having to move alternately on the left and right to escape the currents.
- ***Side to side one feet arms up***
  - *Instructions*. Contract pelvic floor and transversus abdominis keeping them activated throughout the exercise. Maintain trunk and pelvis aligned. Breathe in while bringing the arms over the head with the palms facing each other, shifting the body weight on the right foot, and lifting the left foot. Maintain the position for 3 respiratory cycles. Breathe out and relax the arms along your sides, while shifting the weight in the middle; then, shift the body weight on the left foot and lift the right foot while bringing your arms over your head. Maintain the position for 3 respiratory cycles. Breathe out while returning to the starting position. Repeat at least 3 times.
  - *Imagery*. Imagine being on two stones in a river and having to move alternately on the left and right to escape the currents.

***Hip circle***

***Primary aims***: correct load transfer, monopodalic stance, balance maintenance in monopodalic stance.

***Secondary aims***: Core activation maintained during limb-to-limb load transfer in monopodalic stance, trunk-head alignment maintenance during limb-to-limb load transfer and during flexion-abduction-hip external rotation, coordination between breathing and movement, hip joint mobilization in flexion, abduction and external rotation.

***Position***: standing, backbone in neutral position, feet firmly on the ground and aligned with knees and hips, hands on hips.

***Exercise***:

- ***Hip circle***
  - *Instructions*. Contract pelvic floor and transversus abdominis keeping them activated throughout the exercise. While maintaining the pelvis stabilized, shift the body weight on a leg. Breathing in while lifting the other leg by flexing the knee; breathe out while abducting the leg by externally rotating the hip, and then return to the starting position. Repeat at least 3/5 times on each side.
  - *Imagery*. Imagine the hip joint as a well-oiled cog.

***Side Leg Kick***

***Primary aims***: correct load transfer, monopodalic stance, balance maintenance in monopodalic stance.

***Secondary aims***: Core activation maintained during limb-to-limb load transfer in monopodalic stance, axial backbone alignment maintenance in lateral inclination, dynamic shoulder girdle stabilization, hip and scapula-humeral joint mobilization in abduction-adduction.

***Position***: standing, backbone in neutral position, feet firmly on the ground and aligned with knees and hips, arms at sides.

***Exercise***:

- ***Side Leg Kick 1***
  - *Instructions*. Contract pelvic floor and transversus abdominis keeping them activated throughout the exercise. Breathe out while stabilizing the backbone, leaning the torso on one side and abducting the contralateral leg. Breathing in while returning to the starting position, and then repeat on the other side while breathing out. Repeat at least 2 times on each side.
  - *Imagery*. Imagine picking a fruit from a tree.
- ***Side Leg Kick 2***
  - *Instructions*. Contract pelvic floor and transversus abdominis keeping them activated throughout the exercise. Breathe out while stabilizing the backbone, leaning the torso on one side and abducting the contralateral leg and arm. Breathing in while returning to the starting position, and then repeat on the other side while breathing out. Repeat 2 times for each leg.
  - *Imagery*. Imagine picking a fruit from a tree.

***One leg circle***

***Primary aims***: correct load transfer, monopodalic stance, balance maintenance in monopodalic stance.

***Secondary aims***: Core activation maintained in monopodalic stance, trunk-head alignment maintenance during hip circumduction, coordination between breathing and movement, hip joint mobilization in circumduction.

***Position***: standing, backbone in neutral position, feet firmly on the ground and aligned with knees and hips, arms at sides.

***Exercise***:

- ***One leg circle skimmed***
  - *Instructions*. Contract pelvic floor and transversus abdominis keeping them activated throughout the exercise. Maintain the trunk aligned. While shifting the body weight on one leg, with the toe of the other leg draw circles on the ground by touching the floor. Breathe out, while moving the leg sideways and backwards; breathe in while moving the leg forward. Repeat at least 5 times on each side.
  - *Imagery*. Imagine the leg as a compass drawing a perfect circle.
- ***One leg circle***
  - *Instructions*. Contract pelvic floor and transversus abdominis keeping them activated throughout the exercise. Maintain the trunk aligned. While shifting the body weight on one leg and lifting the other, draw circles slightly off the floor. Breathe out, while moving the leg sideways and backwards; breathe in while moving the leg forward. Repeat at least 3 times on each side.
  - *Imagery*. Imagine the leg as a compass drawing a perfect circle.

***Single leg extensions***

***Primary aims***: correct load transfer, monopodalic stance, balance maintenance in monopodalic stance with change of position.

***Secondary aims***: Core activation maintained during limb-to-limb load transfer in monopodalic stance, trunk-head alignment maintenance during limb-to-limb load transfer in monopodalic stance, coordination between breathing and movement, maintenance of axial backbone alignment while tilting frontally, hip and knee mobilization in flexion-extension, shoulder girdle stabilization, shoulder girdle mobilization in circumduction.

***Position***: standing, backbone in neutral position, feet firmly on the ground and aligned with knees and hips, arms at sides. Activate the transverse and pelvic floor and keep them in this way for the entire duration of the exercise.

***Exercise***:

- ***Superman 1***
  - *Instructions*. Contract pelvic floor and transversus abdominis keeping them activated throughout the exercise. Maintaining the trunk aligned. Breathing out while lifting one leg backwards and tilting the torso forward by keeping the arms at the sides. Breathe in while returning to the starting position. Repeat at least 4 times on each side.
  - *Imagery*. Imagine being Superman flying on the sky.
- ***Superman 2***
  - *Instructions*. Contract pelvic floor and transversus abdominis keeping them activated throughout the exercise. Maintaining the trunk aligned. Breathing out while lifting one leg backwards and tilting the torso forward by keeping the arms stretched forward. Breathe in while returning to the starting position. Repeat at least 3 times on each side.
  - *Imagery*. Imagine being Superman flying on the sky.
- ***Superman 3***
  - *Instructions*. Contract pelvic floor and transversus abdominis keeping them activated throughout the exercise. Maintaining the trunk aligned. Breathing out while lifting one leg backwards and tilting the torso forward by keeping the arms stretched forward. Breathe in/breathe out 3 times while maintaining the position and quickly drawing 3 small circles with the arms, firstly in one direction then in the other. Return to the starting position. Repeat at least 3 times on each side.
  - *Imagery*. Imagine being Superman flying on the sky.
- ***Superman 4***
  - *Instructions*. Contract pelvic floor and transversus abdominis keeping them activated throughout the exercise. Maintaining the trunk aligned. Breathing out while lifting one leg backwards and tilting the torso forward by keeping the arms stretched forward. Breathe in/breathe out 3 times while maintaining the position. Then, move back the leg leaned on the toe while realigning the torso. Perform a lunge by flexing both knees. Return to the starting position without put the foot down on the ground. Repeat at least 3 times on each side.
  - *Imagery*. Imagine being Superman flying on the sky.
