# Supplementary material for: An Internet- and Kinect-Based Multiple Sclerosis Fitness Intervention Training With Pilates Exercises: Development and Usability Study
Source: JMIR Serious Games. 2023 Nov 8;11:e41371. doi: 10.2196/41371 (PMC10666018; doi:10.2196/41371)
Supplement: Multimedia Appendix 2 [file games_v11i1e41371_app2.docx]

**Supplementary Material 2. Additional illustration of the device and implementation**


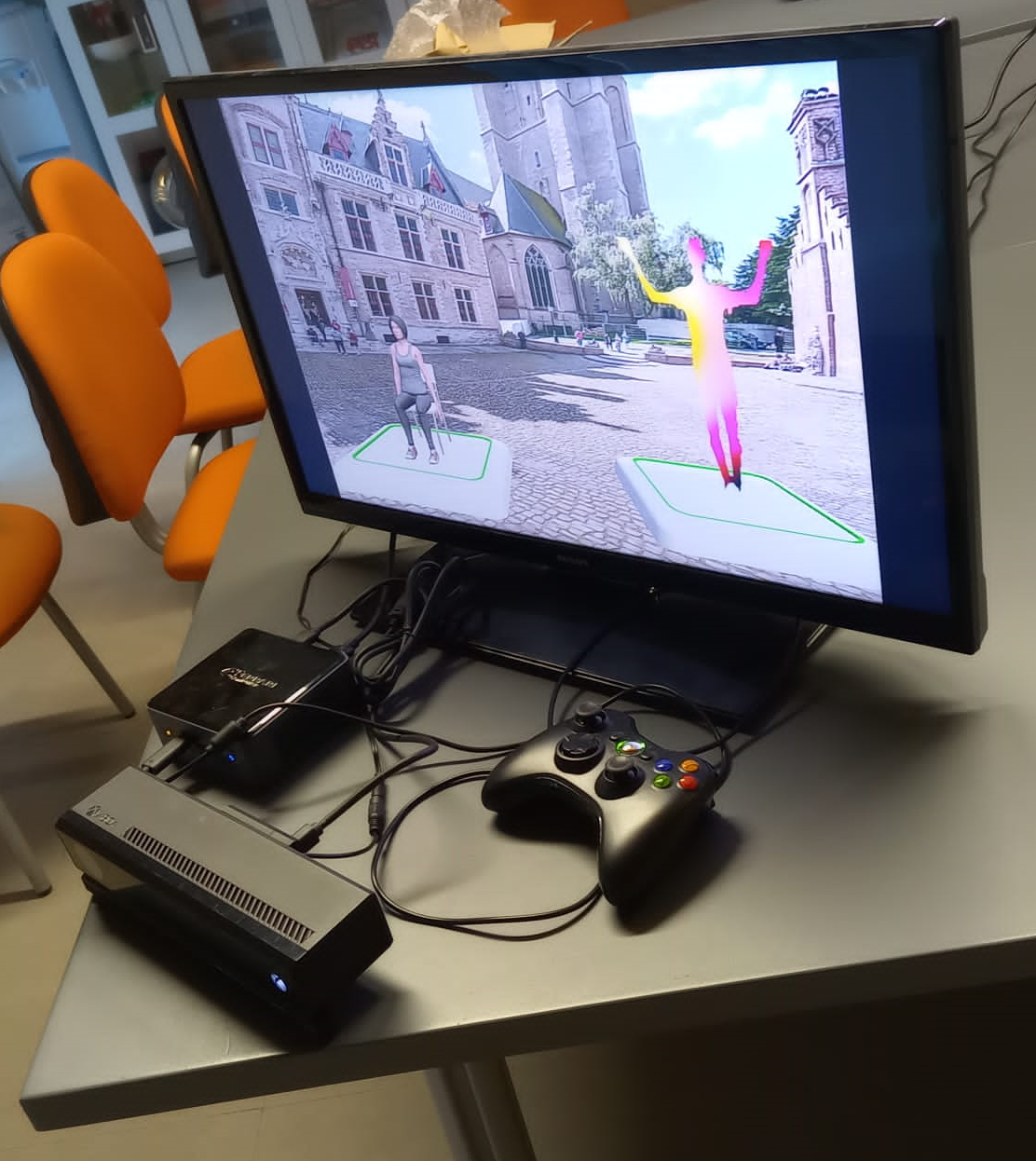


Set-up of MS-FIT. The size of the monitor used in the different phases of the study was 27 tft.

**
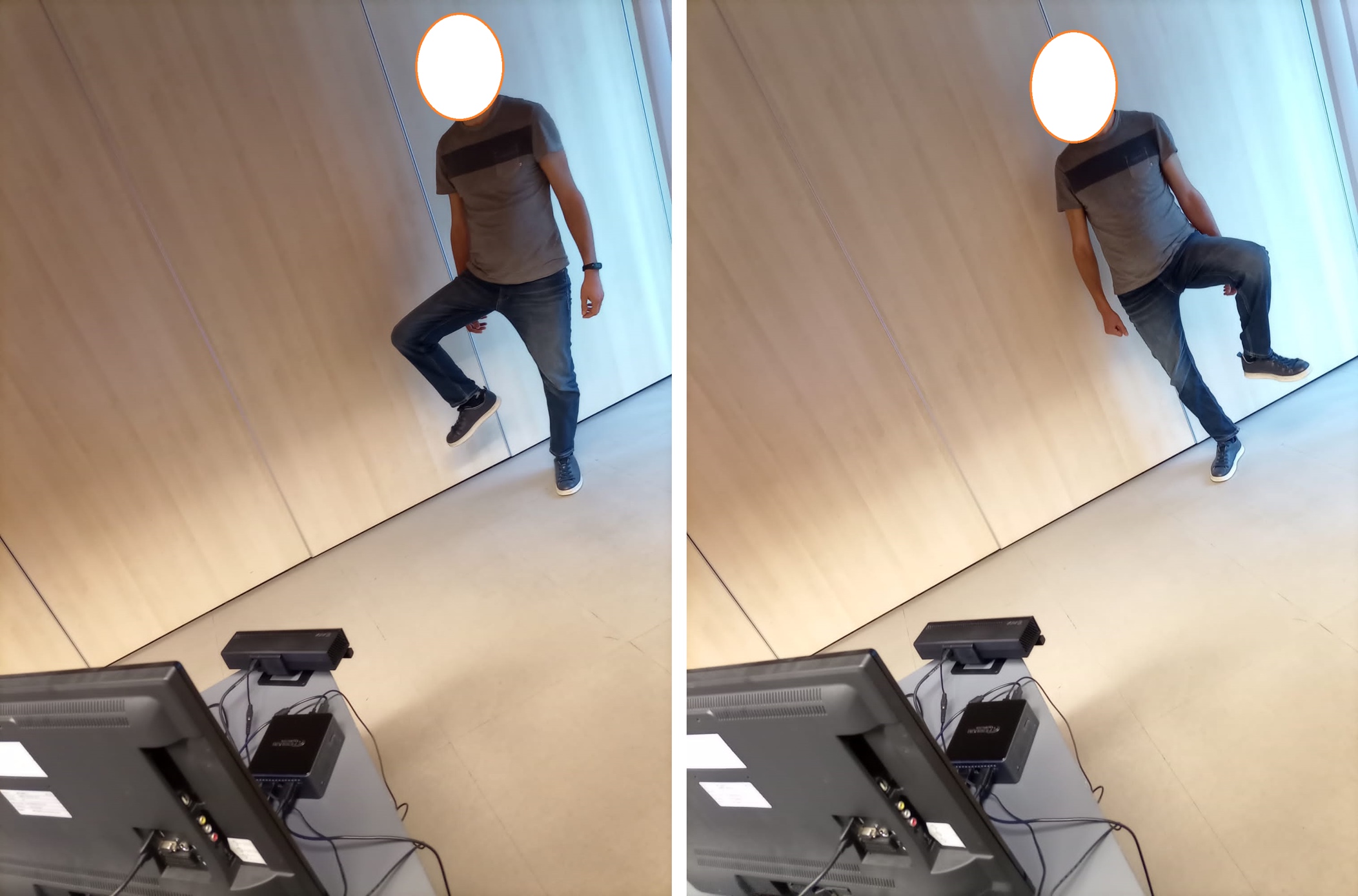
**

Position of the user with respect to the MS-FIT tool (the user are executing the Hip circle exercise)

**
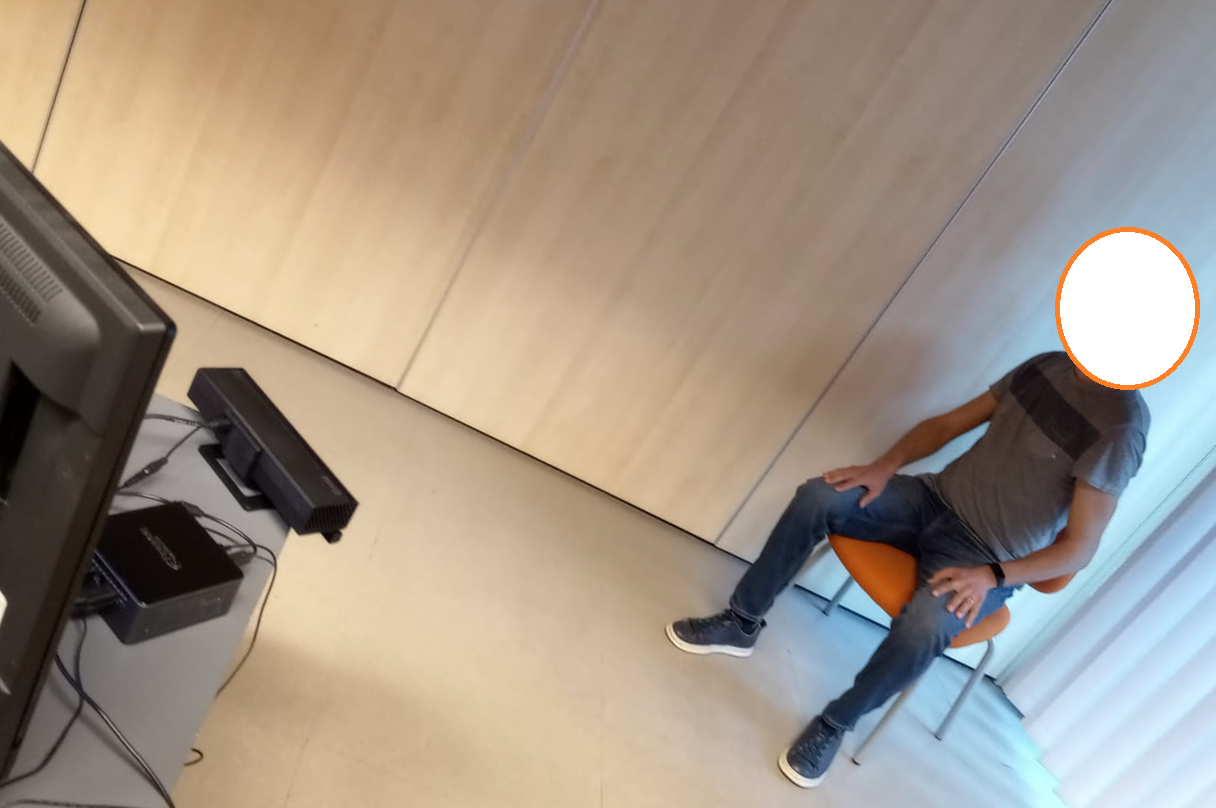
**

Position of the user with respect to the MS-FIT tool (the user are executing the Spine Twin exercise)

**
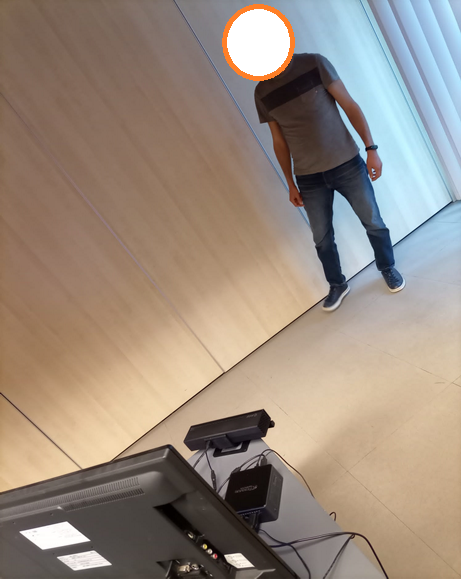
**

Position of the user with respect to the MS-FIT tool (the user are executing the Side to Side exercise)
