# Supplementary material for: An Internet- and Kinect-Based Multiple Sclerosis Fitness Intervention Training With Pilates Exercises: Development and Usability Study
Source: JMIR Serious Games. 2023 Nov 8;11:e41371. doi: 10.2196/41371 (PMC10666018; doi:10.2196/41371)
Supplement: Multimedia Appendix 3 [file games_v11i1e41371_app3.docx]

**Supplementary Material 3. List of participating centres.**

1. Scientific Research Area, Italian Multiple Sclerosis Foundation (FISM), Genoa, Italy
2. Neuroimmunology Unit, Fondazione IRCCS Istituto Neurologico Carlo Besta, Milan, Italy
3. Vita-Salute San Raffaele University, Milan, Italy
4. Department of Neuroscience, Rehabilitation, Ophthalmology, Genetics, Maternal and Child Health, University of Genoa, Genoa, Italy
5. Neurology Unit, IRCSS Neuromed, Pozzilli, Italy
6. Department of Medical Science and Public health, University of Cagliari, Cagliari, Italy
7. Department of Neuroscience, University of Padua, Padua, Italy
8. Department of Basic Medical Sciences, Neuroscience and Sense Organs, University of Bari, Bari, Italy.
9. IRCCS, Don Carlo Gnocchi Foundation, Milan, Italy
10. IRCCS Istituto delle Scienze Neurologiche of Bologna, Bologna, Italy
11. Department of Advanced Medical and Surgical Sciences, University of Campania Luigi Vanvitelli, Naples, Italy
12. Department of Neurosciences, S. Camillo-Forlanini Hospital, Rome, Italy
13. Department of Medical and Surgical Sciences and Advanced Technologies "G.F. Ingrassia", University of Catania, Catania, Italy
14. IRCCS Centro Neurolesi "Bonino-Pulejo", Messina, Italy
15. AISM Rehabilitation Service of Padua, Padua, Italy
16. AISM Rehabilitation Service of Genoa, Genoa, Italy
