# Supplementary material for: An Internet- and Kinect-Based Multiple Sclerosis Fitness Intervention Training With Pilates Exercises: Development and Usability Study
Source: JMIR Serious Games. 2023 Nov 8;11:e41371. doi: 10.2196/41371 (PMC10666018; doi:10.2196/41371)
Supplement: Multimedia Appendix 4 [file games_v11i1e41371_app4.docx]

| **Country** | **Continent** | **City** |
| --- | --- | --- |
| *Belgium* | *Europe* | Bruges, Bruxelles |
| *Canada* | *North America* | Alberta |
| *China* | *Asia* | Beijing, Hong Kong |
| *Cypro* | *Europe* | Nicosia |
| *France* | *Europe* | Paris |
| *Germany* | *Europe* | Berlin, Hannover |
| *Indonesia* | *Asia* | Bali |
| *Italy* | *Europe* | Bologna, Florence, Milan, Rome, Venice |
| *Japan* | *Asia* | Tokyo |
| *Netherlands* | *Europe* | Amsterdam |
| *Portugal* | *Europe* | Lisbon |
| *Spain* | *Europe* | Girona, Madrid |
| *Switzerland* | *Europe* | Brienz |
| *Thailand* | *Aisa* | Bangkok |
| *Turkey* | *Europe* | Adalia, Istanbul |
| *United Arab Emirates* | *Asia* | Dubai |
| *United Kingdom* | *Europe* | London |
| *United States* | *North America* | Grand Canyon, New York |

**Supplementary Material 4. List of cities.**
