# Supplementary material for: An Internet- and Kinect-Based Multiple Sclerosis Fitness Intervention Training With Pilates Exercises: Development and Usability Study
Source: JMIR Serious Games. 2023 Nov 8;11:e41371. doi: 10.2196/41371 (PMC10666018; doi:10.2196/41371)
Supplement: Multimedia Appendix 5 [file games_v11i1e41371_app5.docx]

| **Mission** | **Description** |
| --- | --- |
| *Training* | Number of training days (Level 1: 10, Level 2: 40, Level 3: 80, Level 4: 160) |
| *Traveler* | Number of visited cities (Level 1: 5, Level 2: 15, Level 3: 28) |
| *International* | Number of visited countries (Level 1: 2, Level 2: 4, Level 3: 10) |
| *World* | Number of visited continents (Level 1: 2, Level 2: 3) |
| *Habitual* | Number of training days in the same city (Level 1: 3, Level 2: 8, Level 3: 20) |
| *Methodic* | Number of consecutive training days (Level 1: 3, Level 2: 5) |
| *Rich man* | Earned coins (Level 1: 3000, Level 2: 9000, Level 3: 20000) |
| *Spendthrift* | Spent coins (Level 1: 3000, Level 2: 9000, Level 3: 20000) |
| *Variety* | Exercises performed at least once (Level 1: 10, Level 2: 20, Level 3: 40, Level 4: 48) |

**Supplementary Material 5. List of the missions.**
